# Surgical Cases from Practice

**Published:** 1893-04

**Authors:** C. D. Hurt

**Affiliations:** Atlanta, Ga.


### SURGICAL CASES FROM PRACTICE.

By C. D. HURT, M. D.,

ATLANTA, GA.

### FRACTURE OF THE SKULL.

CASE 1.—White man, aged 48 years ; by trade wheelwright. I went on call to his workshp, about 140 yards from my office. He had been struck about three minutes before. He was on my arrival in an unconscious state, twisting and moving as if he would rise and walk. He had been struck with a heavy wagon spoke, and fell over his bench, his feet catching and his head descending to the floor heavily. I had him placed in a wagon and carried to his home, about 300 yards. After he was put to bed and I examined him, I found a lacerated wound over the left parietal bone, extending antero-posteriorly three and a half inches and through tissues to the bone underneath. Running my finger along the surface of bone, I found a fissure extending in the same line with flesh wound, and from anterior to posterior borders of parietal bone. There was no depression on either side of fracture. The surface of his body was cold and bathed with perspiration. He was very restless, with considerable muttering and occasional convulsions of the left face and arm. I watched him for an hour, after which time a semi-comatose condition set in, pulse growing less frequent. Still an hour later he vomited freely and seemingly reacted from the shock. After awhile he went into a coma, which lasted four days and then gradually subsided. At the same time he had complete hemiplegia, which continued for three weeks and gradually disappeared. About the 20th of January he recovered his speech and the use of his right side so that he undertook light work in his shop, such as trimming and painting buggies.

On the 15th of February he found his right arm becoming more painful, and the result was that he had to suspend his work and was confined at home for several months.

My treatment at first consisted of opening his bowels with croton oil, and after the subsidence of the coma, of giving ten grains each bromide potassium and chloral every four to six hours. During the spring and summer of '84 he had considerable pain in his head, right arm and leg. His right hand was swollen most of the time. I diagnosed fracture of the left parietal bone without any depression, concussion of the brain with extravasation and blood clots on the brain.

*Comments:* The manner of death from injuries to the brain varies. Death may come on immediately when the wound is at the base of the brain and injury done to the respiratory nerve center when patient recovers from the shock. Death may result in a few hours when hemorrhage sets in from the rupture of sinuses or blood vessels in the cranium and consequently overthrow the nervous system. Encephalitis may set in at any period after rallying from the shock and the patient die as the result. More remotely still, paralysis and other evils from injury to the nervous system may prove fatal. In this case we had first a violent shock and concussion. Before he recovered from the shock he had a dozen or more convulsions of the right arm and face. After vomiting and recovering he then had trouble from the extravasation of blood in and upon the brain tissues which probably resulted from cutting of a meningeal artery. When he recovered from the shock he had complete hemiplegia which continued three weeks, and at the end of five weeks he had recovered his power of speech and partial use of his arm and leg. After beginning work in his shop and working for two weeks partial hemiplegia returned and he was again confined to his house. For months he suffered great pain in his head so that he had to resort to the use of anodynes for relief. If there had been no laceration with extravasation of blood in the brain tissues, would he have had both convulsions and coma? There was evidently considerable pressure from blood clots, producing coma.

During the first three weeks of his illness the sphincters were entirely relaxed, and the action of his bowels was involuntary. His mind was considerably impaired and his disposition exceedingly irritable. The whole nature and manner of the man underwent a change, which condition prevails to this day, February 15, 1892, and will probably continue all the rest of his life.

For the past twelve months he has had fits of madness and insanity, making it so unpleasant for his family they have been unable to live with him. He has, of course, been unable to prosecute his work.

NECROSIS OF THE TIBIA FOLLOWING AN INJURY FROM A FALL.

CASE 2.—Aged 20; family history on both sides good. Five brothers and sisters. Called to see her on March in consultation with Dr. B., who then had the case in charge. Found her on bed suffering great pain, high fever, very pallid, nervous and emaciated, with the following history: Two months previous she fell on a brick pavement striking her shin over the tibia, which caused considerable soreness and swelling at the time. One week later she developed high fever with intense pain which seemed to be in the bone. A physician was called and diagnosed rheumatism and treated her for six weeks. The case not progressing satisfactorily he was dismissed and Dr. B. asked to take charge of the case. Upon examination Dr. B. diagnosed necrosis of the tibia, with already quite an accumulation of denuded bone and pus. He proceeded to open the enlargement at the upper third of the tibia from which escaped a large quantity of strumous pus.

After learning the full history of the case and seeing that she was being rapidly exhausted by fever, I advised an immediate operation. We then proceeded at once to amputate at the lower third of the femur. The patient rallied after the operation and all went well until about the ninth day when she developed a case of measles. The feverish condition attending the measles caused the wound to reopen and suppurate, and the healing process was delayed and completed about the fourth week after amputation. She was soon upon her crutches and taking exercise about the house. A cough which developed during her attack of measles continued to trouble her. She died in about six months from some pulmonary trouble which the attending physician called consumption.

### SPINAL ABSCESS AND NECROSIS.

Case 3. Mrs. F. consulted me on the 13th day of January, 1890, as to a trouble with her daughter, one of twins fifteen years old, well developed, healthy ; never had a week's sickness in her life. She began to menstruate at the age of thirteen years, and had been at the usual school duties for the past several years. Mrs. F. reported that for the past week her daughter had frequent and painful micturition and could only empty the bladder after painful and protracted efforts. Her bowels had been regular until one week ago, since which time they could not be moved with free use of cathartics. Pain along the coccyx, rectum and in all the pelvic viscera ; some weakness and soreness in the sacro-lumbar region, but little if any fever, appetite poor, sleep much disturbed. I ordered a mercurial cathartic, and told Mrs. F. that her daughter was threatened with an abscess—just where and when I could not say from the present symptoms. The girl continued at school with no improvement in her condition ; her bowels and bladder still giving her pain. I was then called to see her. I found her in great pain, especially of the bladder and rectum and along the glutei muscles of the left side, an agonizing pain in both feet, described as producing a sensation like that when walking on small fragments of broken glass. I directed her mother to stop her from school and to use sufficient cathartics to secure daily action from the bowels.

My next visit was on the 3d of February, when I found pain as before and extended to the sacro-lumbar junction. Upon a careful examination I found small tumefaction on the left of the spinal column and just above the sacrum, and detected deep fluctuation. I diagnosed pus and called in Dr. Walker who concurred with me, and under an anesthetic administered by him I made an incision large enough to introduce my index finger, and found on exploration about two drachms of cheesy pus under the periosteum covering the dorsal surface of the sacrum. The patient's temperature since my first visit had ranged from 100 to 101½. After emptying the cavity and washing it out with 1-3000 bichloride solution, I applied iodoform dressing. My patient continued to suffer such pain in the pelvic and lower extremities as to require the regular use of hypodermics of morphia. On the 10th of February I enlarged the opening to two inches, and upon introducing my finger found two or three pieces of bone detached from the spinous process of the lower lumbar vertebra; also three distinct sacks of pus from which I emptied 1½ ounces. Believing that this pus came partially from the spinal cavity, I introduced my finger between the vertebræ and found that I could reach the spinal cord. I washed the wound again with antiseptics and dressed with iodoform gauze. The patient carried about the usual temperature with a slight tendency upward with no abatement of pain whatever. I continued to dress the wound twice daily for a week longer. During this time paresis of the bladder necessitated the use of a catheter. Lower bowel totally inactive, would not respond to any cathartic and had to be washed out by enemas. About the 17th of February her bowels begun to discharge involuntarily and without her knowledge. Paralysis of motion now existed in the lower extremities, but the peripheral pain was so intense as to forbid them being handled. She had lost appetite and flesh and was never quiet or comfortable except when under the influence of an andoyne. I continued for ten days to irrigate the wound with mild antiseptics, and at the end of this time extended the incision upward along the spinal column for 3½ inches and found suppuration extending along the entire lumbar vertebra, portions of which were denuded and honey-combed. I then informed Mrs. F. that the disease had progressed so as to make death inevitable, and my patient died exhausted in about thirty-six hours.

This patient brought about her trouble by taking a long and heating walk and then sitting for sometime on very damp ground.

NECROSIS OF THE ENTIRE FIBULA FROM AN INJURY.

CASE 4. Aged 22 years, one of eight children, family history good. When a child a few months old he lived in a malarial district; contracted chills which lasted him twelve months, after which he was always delicate and frail. During all of his life had frequent attacks of headaches. When about eight years of age tonsils became enlarged and remained so for eight years; a little hard of hearing. Exposure to heat in the summer or to cold in winter seemed to produce an attack of headache.

In December, 1889, while clerking in one of our business houses, he went to the assistance of a drayman who was unloading a box of goods. In lifting it from the dray it fell, striking the patient on the breast, then sliding down, it struck with a great deal of force the upper end of the fibula of the left leg and glided very heavily down the entire length of the bone. He complained of lameness and soreness and remained at home two or three days. He then went about his work but continued to complain, and would make an application of liniment before retiring at night. He stated that the leg continued to swell until about the last of February, 1890, when I was called to examine it. On examination I found the leg intensely swollen from knee to ankle, very dark, very painful, and palpation discovered quite an accumulation of pus. His temperature was 102. I diagnosed necrosis of the fibula. The leg was opened its entire length, and passing the finger up and down the outer border of the fibula, found it at least one-half of its thickness along the middle ; two-thirds of its length had sloughed away. After opening and discharging the pus, the entire cavity was washed out with 1 to 4000 solution of bichloride of mercury and the wound packed with iodoform gauze. On the following day patient felt much more comfortable. Temperature 100, appetite, which had been poor, somewhat improved. Ten days after the operation patient began to use crutches, and daily dressings of the wound with iodoform were continued. About the first of April, the wound, which had not healed, began to fill in with a granular fungous growth. In two or three days this growth became so enormous as to separate widely the edges of the incision, and the wound looked as if two inches in width of the cuticle had been dissected away. Pus continued to discharge from about the bone, and an occasional piece of the bone was expelled since the first operation. On consultation it was decided to cut away most of this fungous growth and to pass a drainage tube through the back portion of the limb and encourage free drainage. On the 5th of April he was put under the influence of ether and the operation performed, which gave very great shock. He did not completely rally and died exhausted on the evening of the 6th.

From the time he received the injury until the first operation he had a continued nervous cough without any expectoration. After the operation cough ceased entirely. While the family history was very good, he had a sister to die at the age of seventeen with hydatids of the liver. She had a continued discharge from a fistulous opening just below the border of the ribs for ten months.

### GUNSHOT WOUND OF THE HEAD; AN OUNCE OF BRAIN REMOVED; RECOVERY.

CASE 5.—A boy fourteen years of age, while out in the field hunting with a friend, received the contents of a shotgun discharged a few feet from him. The load struck his head one and a half inches above the right brow, passed in the direction of the temporal bone, removing a portion of both the frontal and temporal bones, leaving a wound in which I laid the index and middle fingers. I found him in a profound coma, with feeble and rapid pulse, his face covered with blood and a mass of brain tissue protruding from the opening in his skull. I removed with my scalpel an ounce of brain denuded and blackened with powder, put on antiseptic dressing and left him, thinking that he would die in a few hours.

The following day I found him semi-conscious, without fever, rather restless. As the dressing was soiled from hemorrhage I renewed it, ordered an enema of warm water to move the bowels, an anodyne of bromide of potash and chloral to be given *pro re nata;* sweetmilk as diet. On the fourth day I discovered a gangrenous condition of wound, the tissues separating from the margin of the bone. I applied the following mixture to the wound, except the brain: Carbolic acid one part, tincture of iodine two parts, glycerine five parts. I reapplied this every second or third day until at the end of a week the wound assumed a healthy granulating appearance.

With very little fever and no unfavorable symptoms he continued to improve until at the end of ten weeks the wound had entirely healed with a cicatricial covering. The injury was received on the 25th day of December, and on the first of April he resumed his school duties with his usual energy and wit.

